# Anti-Diabetic Potential of Plant-Based Pentacyclic Triterpene Derivatives: Progress Made to Improve Efficacy and Bioavailability

**DOI:** 10.3390/molecules26237243

**Published:** 2021-11-29

**Authors:** Michael Oboh, Laurencia Govender, Muthulisi Siwela, Blessing Nkazimulo Mkhwanazi

**Affiliations:** Dietetics and Human Nutrition, School of Agricultural, Earth and Environmental Sciences, University of Kwazulu-Natal, Private Bag X01, Scottsville 3209, Pietermaritzburg 3201, South Africa; 220113325@stu.ukzn.ac.za (M.O.); govenderl3@ukzn.ac.za (L.G.); siwelam@ukzn.ac.za (M.S.)

**Keywords:** anti-diabetic, bioavailability, diabetes mellitus, medicinal plants, oleanolic acid, pentacyclic derivatives

## Abstract

Diabetes mellitus (DM) results from the inability of the pancreas to produce sufficient insulin or weakened cellular response to the insulin produced, which leads to hyperglycemia. Current treatments of DM focus on the use of oral hypoglycemic drugs such as acarbose, alpha-glucose inhibitors, sulphonylureas, thiazolidinediones, and biguanides to control blood glucose levels. However, these medications are known to have various side effects in addition to their bioavailability, efficacy, and safety concerns. These drawbacks have increased interest in the anti-diabetic potential of plant-derived bioactive compounds such as oleanolic and maslinic acids. Although their efficacy in ameliorating blood glucose levels has been reported in several studies, their bioavailability and efficacy remain of concern. The current review examines the anti-diabetic effects of oleanolic, maslinic, asiatic, ursolic, and corosolic acids and their derivatives, as well as the progress made thus far to enhance their bioavailability and efficacy. The literature for the current review was gathered from leading academic databases—including Google Scholar and PubMed—the key words listed below were used. The literature was searched as widely and comprehensively as possible without a defined range of dates.

## 1. Introduction

Medicinal plants, also known as medicinal herbs, have been a significant source of bioactive compounds to treat various diseases. They possess a range of preventative and pharmacological properties such as anti-diabetic, anti-hyperlipidemia, anti-microbial, anti-cancer, anti-hypertensive, antioxidant, and hepatoprotective properties [1,2]. Such complementary medicine is still extensively practiced today, especially by the African people [3,4]. About 80% of South Africans and the world’s population still rely on medicinal plants for the management of different ailments due to their availability, low cost, potential efficacy, low toxicity, and transient side effects compared to synthetic medications [5,6]. Interestingly, medicinal plant-derived products and biopharmaceuticals are now used by western societies [7]. Their use through much of human history in managing physical and mental ailments has been an outcome of the invaluable knowledge and experiences acquired by natives through cultural practices. 

Bioactive compounds, such as terpenoids, alkaloids, carotenoids, and flavonoids [8,9] present in medicinal plants exert their therapeutic effects by inhibiting α-amylase, α-glucosidase, pancreatic lipase, and scavenging free radicals [10,11,12]. Furthermore, several other bioactive compounds have been isolated from medicinal plants such as *Indianthus virgatus, Mangifera indica, Allium sativa, Olea europaea,* and *Centella asiatica*. These isolated compounds such as oleanolic acid, ursolic acid, betulinic acid, and maslinic acid possess anti-diabetic activities [1,13,14].

Pentacyclic triterpenes and their derivatives are among the most widely researched anti-diabetic compounds. Oleanolic acid has shown a dose-dependent inhibitory activity on α-glucosidase and α-amylase [15]. Further study revealed that the anti-diabetic effects of oleanolic acid were through the inhibition of salivary α-amylase and pancreatic lipase activities in individuals with impaired fasting blood glucose levels. Oleanolic acid has been reported to increase insulin response, inhibition of protein-tyrosine phosphatases to improve insulin response, and glucose uptake [16]. Findings have indicated that the hypoglycemic activities of maslinic acid and related pentacyclic triterpenes exerted their effects through the inhibition of glycogen phosphorylase (GP) and protein tyrosine phosphatase 1B (PTP1B) [17]. Corosolic acid exhibits anti-diabetic effects in humans by reducing plasma glucose levels after a glucose challenge [18]. The current review aims to provide a comprehensive list of medicinal plants that have been found containing appreciable concentrations of pentacyclic derivatives possessing anti-diabetic effects, as well as give an up-to date account and evaluation of research progress achieved to improve the efficacy and bioavailability of these bioactive compounds (pentacyclic derivatives).

## 2. Methods of Data Collections 

This current study is review, which used data from experimental findings and clinical trials on the anti-diabetic activity, bioavailability, and efficacy of pentacyclic triterpenes. This was obtained using search engines such as Google Scholar and PubMed. To conduct a comprehensive review on the medicinal plants, this review was not limited by the time of publication or a selected period. This in part was due the paucity of clinical trial studies on triterpenes which would be limited by a time range. Duplicates were removed and abstracts were read for relevance. Thereafter all irrelevant journal articles were removed. Two researchers with experience in the topic then read through the remaining articles and book chapters and excluded all articles and book chapters that did not meet the research criteria. The search was done using keywords such as anti-diabetic, pentacyclic triterpenes, bioavailability of triterpenes, pentacyclic triterpenes bioavailability, efficacy of triterpenes, nanoemulsions, medicinal plants, *Syzygium aromaticum* clove, *Xylopia aethiopica*, *Gypsophila oldhamiana*, *Phyllanthus amarus*, oleanolic acid, maslinic acid, asiatic acid, maslinic acid derivatives, corosolic acid, corosolic acid derivatives, ursolic acid, diabetes mellitus, triterpene nanoemulsions, asiatic acid and glycogen phosphorylase, corosolic acid on weight gain, and asiatic acid derivatives. Data search was done in English, although no restrictions were included. All articles which met the selection criteria were selected and used to conduct the literature search. 

## 3. Discussion 

### 3.1. Phyto-Derived Triterpenes for the Management of Diabetes Mellitus

#### 3.1.1. Triterpenes

**Figure 1:**
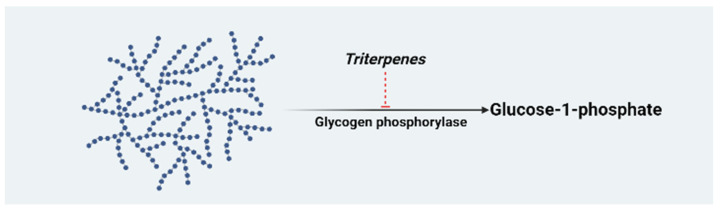
Mechanism of action of maslinic acid as a glycogen phosphorylase inhibitor. The triterpene play a rate limiting step in glycogenolysis by catalyzing the breaking down of glycogen into glucose-1-phosphate by breaking the α-1,4-glycosidic bond [35] (This diagram was creacted with BioRender.com).

Triterpenes belong to the largest group of biologically active plant products known as terpenes. They are primarily found on plant surfaces such as stem bark or leaf and fruit waxes [19]. Their concentration in different plants is affected by various factors, such as different seasons, species, and geographical location [20]. Chemically, triterpenes contain six isoprene units [(CnHn)_6_: C30] and can exist as monocyclic, dicyclic, tricyclic, tetracyclic, and pentacyclic derivatives. Many triterpenes occur in free form, while others exist as glycosides (saponins), esters, or both. There are about 100 different skeletal types of cyclic triterpenes, which form the majority of natural triterpenes [21]. Triterpenes are formed through the isoprenoid pathway by the formation of squalene (the simplest form of triterpene) from two molecules of farnesyl pyrophosphate joined tail-to-tail [22]. Naturally, triterpenoids exist as pentacyclic or tetracyclic structures, and the pentacyclic triterpenes can be grouped into lupanes (betulin, lupeol, and betulinic acid), oleananes (oleanolic and maslinic acids), and ursanes (asiatic, ursolic, and corosolic acids) [14]. Triterpenes are lipophilic in nature, with a lipophilic component (triterpenoid aglycone) attached to a hydrophilic component. They can be acylated or glycosylated (triterpenoid saponins) [23]. The anti-glycation activity of twelve triterpenoid saponins isolated from the root bark extract of *Aralia taibaiensis* was evaluated to corroborate the anti-diabetic activity of the crude extract [24]. Diabetes can result from chronically high levels of glucocorticoids through the enzymatic action of 11β-hydroxysteroid dehydrogenase type 1 (11β-HSD1). Some triterpene saponins isolated from *Barringtonia acutangula* have shown anti-diabetic activity by interacting with the active site amino acid residues of 11β-HSD1 and targeting other protein molecules involved in insulin insensitivity pathways [25]. Considerable attention has been given to triterpenes as potential sources for the development of active pharmacological agents. This is due to several bioactivities of phyto-derived triterpenes, and the exponential increase of commonly known incurable non-communicable diseases such as DM. Several reports have documented the pharmacological activities of triterpenes, which include anti-diabetic, anti-platelet aggregation, and anti-inflammatory [26], anti-hyperlipidemia [27], anti-microbial [28], and anti-cancer activities. The anti-diabetic activity of *Aesculus turbinata* mediated through the inhibition of glucose absorption was attributed to the presence of triterpenoid saponin [29]. Cloves from *Syzygium aromaticum* possess a wide range of medicinal properties such as antioxidant, anti-diabetic, and anti-microbial activities attributed to the presence of some bioactive constituents such as eugenol, steroids, tannins, alkaloids, flavonoids, hydroxycinnamic acids, and oleanolic and maslinic acids [30,31]. Additionally, the anti-hyperglycemic activities of oleanolic and maslinic acids derived from *Syzygium aromaticum* cloves have been reported [32,33,34]. Triterpenes lower blood glucose concentrations in part by inhibiting the conversion of glycogen phosphorylase to glucose-1-phosphate. Glucose-1-phosphate is later converted to energy in the muscles and glucose-6-phosphatase in the liver, thus suppressing glucose release. Therefore, the inhibition of glycogen phosphorylase is a good therapeutic target for type 2 DM (Figure 1). 

#### 3.1.2. Diabetes and Biochemical Targets of Triterpenes

Diabetes mellitus is a metabolic disease caused by insufficient insulin secretion and/or insulin resistance, leading to high blood glucose levels [14]. Insulin insensitivity and deficiency compromise the blood regulatory mechanisms in patients with diabetes, thereby leading to hyperglycemia—a characteristic feature of DM [36]. Uncontrolled hyperglycemia can result in diabetic complications such as nephropathy, neuropathy, retinopathy, diabetic wound, stroke, and cardiovascular diseases [37]. As a result, treatments involving pharmaceuticals primarily focus on controlling hyperglycemia by increasing insulin sensitivity, insulin secretion, and through the inhibition of carbohydrate digestive enzymes [38]. Several studies, both in vitro and in vivo, have shown the anti-diabetic activities of triterpenes, including pentacyclic triterpenes [39]. Furthermore, these compounds have been reported to have high antioxidant activities that help prevent the accumulation of advanced glycated end-products, which have been implicated in the development of diabetic complications [39].

**Figure 2:**
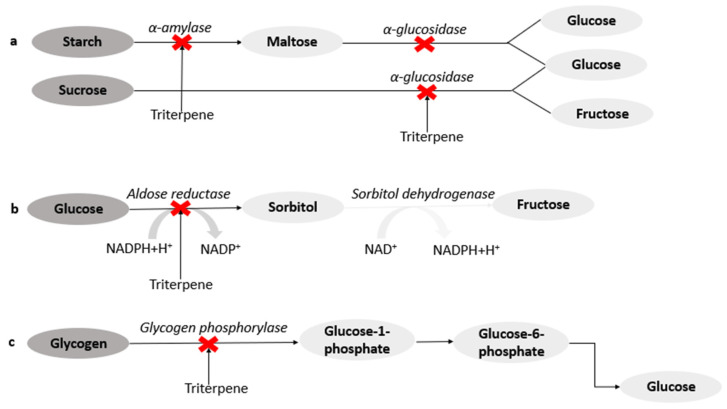
Mechanism of action of 2,3-seco-20(29)-Lupene-2,3-dioic acid, α-amyrin-3O-β-(5-hydroxy) ferulic acid, and pistagremic acid (**a**); maytenfolic acid, 3β,22α-dihydroxyolean-12-en-29-oic acid, and kotalagenin 16-acetate (**b**); corosolic, maslinic, oleanolic, asiatic, tormentic acids (**c**).

Pentacyclic triterpenes exhibit anti-diabetic effects through several target pathways. Some triterpenes, 2,3-seco-20(29)-Lupene-2,3-dioic acid, α-amyrin-3O-β-(5-hydroxy) ferulic acid, and pistagremic acid, obtained from the leaves and twigs of Fagus hayatae (Fagaceae), the root bark of Euclea undulate (Ebenaceae), and galls of Pistacia chinensis var. integerrima, respectively, have been reported to exhibit anti-diabetic activities through the inhibition of α-glucosidase (Figure 2a) [40,41,42]. Docking studies on pistagremic acid further revealed that the compound possessed the specific shape and size for the formation of a hydrogen bond at the active site of α-glucosidase [42]. The α-glucosidase inhibitory activity of euscaphic acid (IC_50_: 0.67 mM) and p-coumaroylursolic acid (IC_50_: 0.62 mM) obtained from the roots of Sanguisorba tenuifolia (Rosaceae) compared to acarbose (IC_50_: 0.79 mM) have been reported [43]. The synergistic effect of ursolic and oleanolic acids extracted from Phyllanthus amarus (Euphorbiaceae) on porcine pancreatic α-amylase, had an IC_50_ value of 4.41. µM [44]. In the diabetic state, the accumulation of sorbitol in tissues such as the retina, lens, kidney, and nerves, resulting from the action of aldose reductase, an enzyme that converts glucose to sorbitol in the polyol pathway, may lead to diabetic complications [45]. Some triterpenes such as maytenfolic acid, 3β,22α-dihydroxyolean-12-en-29-oic acid, and kotalagenin 16-acetate extracted from the roots of Salacia oblonga, were found to inhibit aldose reductase (Figure 2b) [46].

A ursane-type of triterpene and triterpenic acids isolated from leaves of Rhododendron brachycarpum and leaves and stems of Phoradendron reichenbachianum have been reported to form hydrogen bonds at the PTP1B (a negative regulator of insulin signalling pathway) binding sites, thereby increasing insulin response [47]. The inhibition of GP has been reported as a potential target for the control of hyperglycemia. Oleanolic acid and hederagenin isolated from Gypsophila oldhamiana have been reported as some of the most active inhibitors of GP. This activity was attributed to the presence of a hydroxyl group at C-3 and CH_2_OH at C-23 in the oleanane structure [48]. Furthermore, corosolic, maslinic, asiatic, and tormentic acids obtained from Potentilla biscolor, have shown inhibitory activity on GP (Figure 2c) [49,50].

Docking studies have been carried out on several triterpenes to determine their binding modes. Ficusonolide, a triterpene lactone isolated from Ficus foveolata, showed strong interaction when docked into the active sites of PTP1B, dipeptidyl peptidase-IV, α-amylase, and α-glucosidase [51]. Another computational study reported lupeol’s allosteric inhibitory activity on PTP1B. The hydrophobic nature of lupeol (with one hydroxyl group) has been suggested to play a critical role in its interaction by targeting the allosteric hydrophobic sites of PTP1B [52]. The protein–ligand interactions of oleanolic, ursolic, moronic, and morolic acids were adjacent to the active sites of PTP1B and stabilized by hydrogen and Van der Waals bonds [53]. Oleanolic acid from Xylopia aethiopica fruit showed a weak binding affinity for α-glucosidase and α-amylase, possibly due to the absence of hydrogen bond interaction. However, this may not affirm the weak inhibitory activity of oleanolic acid on the two enzymes, because computational analysis sometimes differs from in vitro studies [54].

#### 3.1.3. Anti-Diabetic Activities of Triterpenes

The discovery of potent bioactive compounds from plant materials has gained attention as different groups of chemical compounds such as triterpenes have shown multiple pharmacological activities including anti-diabetic activity. Malviya [55] has previously reported the anti-diabetic efficacy of compounds such as terpenoids obtained from plants compared to conventional anti-diabetic agents. Furthermore, the blood glucose-lowering effect of triterpenoids is mediated through the regulation of glucose transport, glucose uptake, glucose absorption, insulin secretion, and increased glycogen synthesis, thereby ameliorating diabetic vascular dysfunction, diabetic retinopathy, and kidney disease [32,56,57].

Triterpenoids isolated from *Scleroderma aurantium* (3,25-Dihydroxy-22-acetoxyl-lanosta-8,23-diene) and *Agrimonia pilosa* have been reported to exhibit hypoglycaemic effects by scavenging free radicals and inhibiting α-glucosidase, thereby preventing glucose absorption and oxidative stress which has been implicated in the pathogenesis of diabetes mellitus [58,59]. The anti-diabetic activity of *Calotropis procera* mediated through the inhibition of pancreatic lipase activity has been attributed to the presence of terpenoid in the root extract [60]. Ursolic and oleanolic acids isolated from dried stems of *Bouvardia terniflora* have been reported to exhibit blood glucose-lowering effects in normal and alloxan-diabetic mice [61]. Furthermore, a triterpene isolated from *Viburnum odoratissimum* has been reported to stimulate glucose absorption in insulin-resistant HEPG2 cells, thereby preventing hyperglycemia [62].

The neuroprotective effect of oleanane-type triterpenoid extracted from *Momordica cymbalaria* root was investigated in diabetic peripheral neuropathy. The triterpenoid ameliorated neuronal degeneration and showed significant antioxidant activity by stimulating insulin sensitivity in streptozotocin (STZ)-induced diabetic rats, thereby enhancing cellular glucose uptake [63]. The authors of [64] reported the hypoglycemic activities of oleanolic and maslinic acids via the inhibition of α-amylase, α-glucosidase, sucrase, and by regulating the activities of glucose transporters. Ursolic acid has been reported to show anti-diabetic activity by facilitating glycogen storage, glucose utilization in diabetics, and by regulating the activities of α-amylase and α-glucosidase [56,65]. A previous study by [32] reported the ameliorative effect of maslinic acid on diabetic renal disease by controlling blood glucose levels and altering the pathway involved in the formation of advanced glycation end products (AGEs).

Many triterpenes such as oleanolic acid extracted from *Momordica charantia* have shown significant anti-diabetic, antioxidant, and anti-hyperlipidemic activities in rats through the improvement of β-cell regeneration, glucose utilization, and inhibition of carbohydrate metabolism enzymes [66]. Fruit extracts of *M. charantia* showed a slight hypoglycemic effect on nine type 2 diabetic patients without a significant change in insulin levels [67]. One hundred diabetic patients who were administered *M. charantia* homogenized vegetables showed improvement in blood glucose control. Furthermore, the postprandial and fasting blood glucose levels of thirty diabetic patients were significantly reduced after the administration of two tablets of *M. charantia* for one month (three times daily) [68]. Clinical application of *Cyclocarya paliurus*’s extract, a plant with oleanane- and ursane types of pentacyclic triterpenes, showed significant hypoglycemic effect when administered to diabetic patients for four weeks (three times daily) [69]. In vivo investigations on the extract have also shown inhibitory effects on α-glucosidase and lipase. The leaves extract of *Gymnema sylvestre*, rich in triterpenes, was given for 20 months as a supplement (400 mg daily) to 22 diabetic patients using conventional oral hypoglycemic drugs. Due to the significant improvement in blood glucose levels and higher insulin levels, five patients suspended the use of conventional drugs and continued only with the extract [70].

However, the clinical application of triterpenes remains limited due to solubility, stability, bioavailability, and efficacy issues. Although researchers have explored ways to improve the dissolution and efficacy of these compounds by synthesizing natural chemical derivatives. Nonetheless, the biological activity and efficacy of these compounds is a complex process, which considers the bioavailability at the site of action—a critical problem that remains a challenge in drug development. In this regard, the synthesis of nanoemulsions, an oil-based delivery system, may provide a potent mechanism for enhancing the solubility, absorption, an =d distribution of these inherent hydrophobic compounds [71].

#### 3.1.4. Triterpenes Nanoemulsion in the Management of Diabetes Mellitus

The clinical application of several bioactive compounds including triterpenes found to have medicinal properties such as anti-diabetic, anti-cancer, anti-inflammatory, antioxidant, hepatoprotective, neuroprotective, and nephroprotective activities have been limited due to poor solubility and low bioavailability [72]. Hence, the pharmacokinetic parameters such as solubility, stability, and bioavailability of several triterpenes with anti-diabetic activities can be improved by utilizing a suitable nanocarrier for improved therapeutic efficacy.

The anti-diabetic activity of Gymnemic acid, a triterpenoid glycoside found in *Gymnema sylvestre* has been reported by [73]. Although the mechanism of action includes the inhibition of intestinal glucose absorption, lowering glucose, and increasing insulin levels in diabetic patients’ plasma [74], its pharmacological actions are limited by its low solubility in an aqueous solution. To improve the efficacy of glipizide, a nanosuspension was synthesized and evaluated on STZ-induced rats. The glipizide nanoemulsion showed significant blood glucose-lowering effects in the rat model [75].

In another study, ursolic acid loaded in nanoparticles demonstrated significant dose-dependent anti-diabetic activity by facilitating glucose uptake through the synthesis of glucose transporter isoform 4 (GLUT4) [76,77]. Insulin’s capacity to control blood glucose levels through the regulation of gluconeogenesis and glycogen synthesis is compromised by high levels of lipids and triglycerides in the liver [77]. In the same study, ursolic acid-loaded nanoparticles exhibited significant anti-hyperlipidemic activity, thereby ameliorating insulin resistance. The poor solubility and fluctuating bioavailability of betulin, a known naturally occurring anti-diabetic triterpene, led to the evaluation of betulin-loaded nanoparticles. The betulin nanoparticles increased the bioavailability and in vivo anti-diabetic activity of betulin compared to the natural compound [78].

The medicinal properties of glycyrrhizin, a triterpenoid saponin found in Glycyrrhiza plants, have been determined by many researchers as reported by [79]. Glycyrrhizin was loaded in nanoparticles to improve its pharmacological properties. When compared to metformin, a conventional anti-diabetic drug, glycyrrhizin loaded in nanoparticles showed considerable anti-diabetic and anti-hyperlipidemic activities in type 2 diabetic rats [80]. In another investigation, thymoquinone-loaded nanoparticle was combined with glycyrrhizin-loaded nanoparticles to see how they compared to the single formulation [77,81]. Significantly, the combined formulations’ in vivo anti-diabetic efficacy was improved.

### 3.2. Oleanolic Acid 

**Figure 3:**
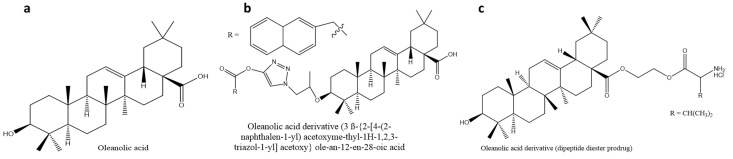
Chemical structure of (**a**) oleanolic acid and its derivatives (**a**) (3β-{2-[4-(2-naphthalen-1-yl) acetoxymethyl-1H-1,2,3-triazol-1-yl] acetoxy} olean-12-en-28-oic acid, (**c**) dipeptide diester prodrug. Derivative (**b**) was synthesized through the addition of bulky hydrophobic groups at carbon-3, which increased antidiabetic properties through glycogen phosphorylase inhibition. Derivative (**c**) synthesized through carbon-28 modification to target peptide transporter 1, thus improving bioavailability.

Oleanolic acid (3β-hydroxy-olean-12-en-28-oic acid) is a natural product from the oleanane group found in a variety of plants in the form of a free acid or triterpenoid saponin [15]. Oleanolic acid is prevalent in the Oleaceae family, and the olive (Oleae europaea), the plant species after which the compound was named, has been the principal source of commercial oleanolic acid [82,83]. This pentacyclic triterpenoid has been isolated from more than 1620 foods and medicinal plants [82,84]. The efficacy of oleanolic acid to prevent acute chemically-induced liver damage, fibrosis, and cirrhosis caused by chronic liver disorders has established its hepatoprotective activity [85]. This compound (Figure 3a) is used in China as an over-the-counter oral drug for the management of liver diseases [86,87]. Furthermore, the authors of [87] reported that oleanolic acid showed antioxidant properties by elevating the expression of thioredoxin peroxidase and catalase and increasing the synthesis of glutathione—an endogenous antioxidant—through a direct chemical reaction with free radicals.

Interestingly, oleanolic acid intake does not result in the production of fat cells, making it a better option than other anti-diabetic medicines, which are typically adipogenic [88]. A prior study investigated the anti-diabetic effects of oleanolic acid and metformin when used separately, compared with their combined effect. Compared to monotherapy, the synergistic impact resulted in a considerable drop in blood glucose levels and enhanced liver function in diabetic mice [88]. Because insulin resistance is a central trait of type 2 DM, oleanolic acid therapy increased insulin sensitivity by boosting the expression of insulin receptor and glucose transporter proteins in HepG2 cells [89]. The authors of [87] reported that treating obese diabetic rats with oleanolic acid for two weeks increased insulin sensitivity, reduced gluconeogenesis, and reduced liver and body weight.

There has been increased interest in the medicinal properties of derivatives of oleanolic acid due to their several biological activities, including glucose-lowering effects in animal models, antioxidant, anti-inflammatory, anti-cancer, and hepatoprotective effects [84,90]. The anti-diabetic effect of novel oleanolic acid derivatives (Figure 3b, Table 1) was investigated, and one, designed by [91], showed significant inhibitory activity on GP and improved glucose absorption. Because GP catalyzes the conversion of glycogen to glucose, inhibiting the enzyme can lower blood glucose levels by lowering hepatic glucose synthesis. The dihydroxy-olide derivative of oleanolic acid has shown anti-diabetic activity through the inhibition of α-glucosidase. 

The pharmacokinetic properties of two amino acid/dipeptide diester prodrugs containing a propylene glycol-linked to oleanolic acid were compared with ethylene glycol-linked amino acid/dipeptide diester prodrugs of oleanolic acid. The study showed that part of the propylene glycol-linked amino acid/dipeptide (Figure 3c, Table 1) had increased permeability, stability, and bioavailability [92,93]. The authors of [92] further reported the improved solubility and bioavailability of oleanolic acid in vitro and in vivo with reduced cytotoxicity when stabilized with nanosuspensions compared to the natural drug. Furthermore, the solubility of oleanolic acid and five of its derivatives (prodrugs) after oral administration to rats was evaluated to be 0.012, 25, 33, 3.7, 3.1, and 12 µg/mL, respectively [92]. When compared to oleanolic acid, the prodrugs appeared to be more aqueous soluble.

#### Clinical Studies on Oleanolic Acid and Its Derivatives

**Figure 4:**
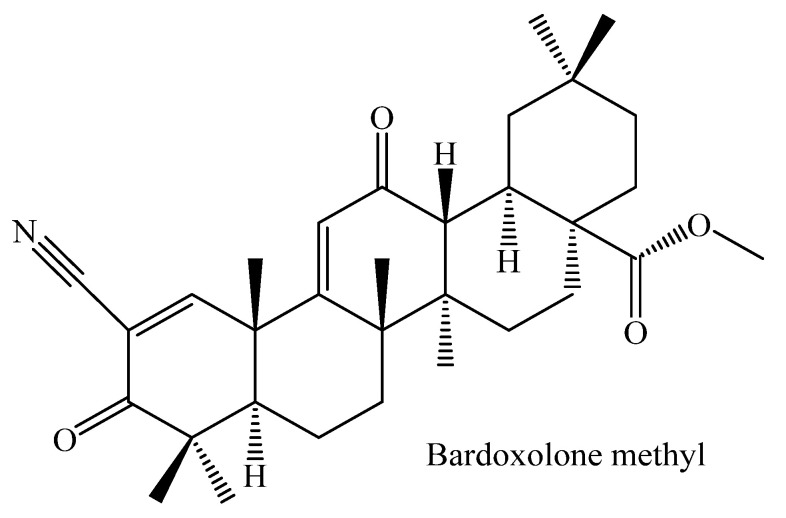
Chemical structure of bardoxolone methyl, a derivative of oleanolic acid synthesized by modifying the Carbon -3 hydroxyl, the ring-C double bond, and the Carbon-28 carboxylic acid.

It is important to note that clinical trials have been carried out using triterpenes as parent compounds. To date, none of the triterpene derivatives that inhibit glycogen phosphorylase inhibitors have gone through to human clinical trials. A clinical trial conducted in China demonstrated that the administration of OA on hyperlipidemic patients for four weeks three times a day lowered total cholesterol, triglycerides without affecting HbA1c (%) and insulin fasting insulin [98]. OA was also shown to improve insulin resistance and regulated glucose metabolism [98]. This made OA a promising agent for fatty liver disease, which often presents before the manifestation of type diabetes mellitus. One study limitation is that the dose of OA was not specified, and the formulation is not precise as OA is known not to dissolve properly in water [98]. A BEACON, randomized control trial, was conducted on 2185 type 2 diabetic patients with stage 4 chronic kidney disease which was estimated by glomerular filtration rate (GRF) and on the placebo group [99]. The diabetic patients with kidney disease received OA derivative bardoxolone methyl [CDDO-methyl Ester (20 mg)] daily. Bardoxolone methyl (Figure 4) is synthesized by modifying the Carbon -3 hydroxyl, the ring-C double bond, and the Carbon-28 carboxylic acid [100]. The clinical trial results indicated a slight significant decrease in the systolic blood pressure compared to the placebo group. However, the mean diastolic pressure increased when compared with the baseline, while the blood pressure decreased in the placebo group. The bardoxolone methyl group significantly increased the heart rate, which was undesirable and resulted in the termination of the clinical trial [99]. The study results also indicated that bardoxolone had no effects on end-stage renal failure and death related to the cardiovascular effects after four weeks of treatment [99]. 

Another clinical trial was conducted to investigate the safety of bardoxolone methyl (20 and 80 mg) on 179 healthy subjects by evaluating the corrected QT intervals (QTc). On the contrary, administration of bardoxolone methyl for six consecutive days showed no cardiovascular adverse effects as it did not significantly affect the QT intervals indicating no adverse cardiovascular effects. A recent study by Lewi et al. performed secondary data analysis of the BEACON clinical trial to investigate the effects of bardoxolone methyl on the hepatic enzymes deLe [101]. The study findings showed increased aminotransferase (ALT), aspartate aminotransferase (AST), and gamma-glutamyl transferase after four weeks; however, there was a downward trend after 48 weeks. Cell culture studies were done to elucidate further whether the increase in hepatic enzymes was due to toxicity. The study showed that ALT and AST isoforms’ mRNA expression was associated with Nuclear factor-erythroid factor 2-related factor 2 (NRF-2). NRF-2 expression is linked with cellular protection as it induces antioxidants, anti-inflammatory effects, thus providing hepatoprotection instead of hepatotoxicity [102]. Taken together, normal QT prolongations and NRF-2 expression may be the markers of toxicity to be considered for drug development. Furthermore, clinical studies are done globally to investigate Alport syndrome in type 2 diabetic patients with chronic kidney disease (CKD) in Japan. 

### 3.3. Maslinic Acid (MA)

**Figure 5:**
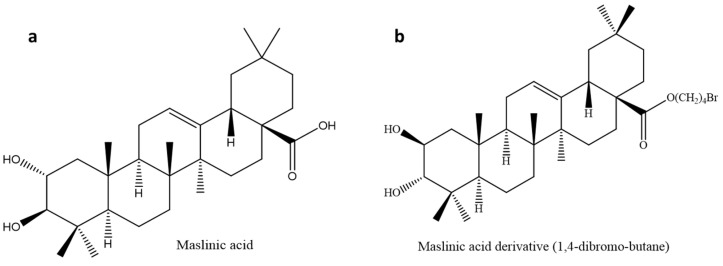
Chemical structure of (**a**) maslinic acid and maslinic acid derivative (1,4-bromo-butane). The maslinic acid derivative (**b**) was synthesized by modifying Carbon 28, thus improving anti-glycogen phosphorylase properties.

Maslinic acid is a natural oleanane-type pentacyclic triterpenoid found primarily (approximately 80%) in the fruits and leaves of *Olea europaea L*. and in *Cornus kousa*, *Ulmus pumila*, and *Junillia Aspera* [103,104,105,106]. Maslinic acid (Figure 5a) has shown anti-diabetic, anti-inflammatory, anti-microbial, anti-viral, and antioxidant properties [107,108,109,110]. This triterpene has been studied to exhibit anti-diabetic effects in muscle and liver cells of rats by inhibiting GP and stimulating tissue growth [111,112]. The authors of [113] also reported that the anti-diabetic of maslinic acid activity was through the inhibition of hepatic GP to bring about a reduction in blood glucose levels. 

Maslinic acid has previously been reported to have anti-cancer activities by inducing apoptosis through maslinic acid-mediated reactive oxygen species [112]. The oral administration of maslinic acid resulted in weight loss and reduced food intake, resulting in improved insulin sensitivity and blood glucose levels in mice [114]. Furthermore, a study showed that the oral administration of maslinic acid resulted in poor bioavailability, which was attributed to its hepatic first-pass metabolism or poor gastrointestinal tract absorption [115].

#### 3.3.1. Maslinic Acid Derivatives for Increased Inhibition of Glycogen Phosphorylase

Glycogen phosphorylase is an important enzyme that catalyzes the breakdown of glycogen to glucose for energy production [116]. Maslinic acid is a moderate inhibitor (IC_50_: 30 µM) of GP; this had more research geared towards synthesizing derivatives to improve its efficacy and potency. A study identified various active sites for maslinic acid (MA), with carbon-28 being the active site corresponding to GP inhibition [94]. Based on this discovery, a series of derivatives were synthesized and investigated against rabbit glycogen phosphorylase. Maslinic acid and most triterpenes are hydrophobic in nature, making them difficult to dissolve in water. A series of hydrophilic compounds were added to carbon 28 to enhance the hydrophobicity of MA. This led to a significant decrease in potency with IC_50_ values of 153, 51, and 580 µM, most of which had lower efficacy than caffeine (IC_50_: 144 µM) [94]. This discovery led to the addition of hydrophobic functional groups on carbon 28 to synthesize 1,4-dibromo-butane (Figure 5b, Table 1), which improved the potency of MA (IC_50_ of 7 µM). Molecular docking studies lead to a conclusion that MA binds to the inhibitor site I of the GP enzyme. Much more in vivo studies are needed for the evaluation of hydrophobic compounds on in vivo animal models. To improve the bioavailability of these hydrophobic compounds, the synthesis of nanoparticles may be a viable route. 

#### 3.3.2. Clinical Studies on Maslinic Acid 

There is a surprising lack of human clinical trials that investigate anti-diabetic properties of MA. Most clinical trials evaluated the effects of MA on arthritis, inflammation, its use as a nutritional supplement. An open-label clinical trial was done on the elderly with an average age of 70.7 ± 10.1 to investigate the effects of daily consumption of MA (30 mg) on chronic knee pain [117]. The daily administration of MA improved the quality of life of the elderly by preventing knee pain, possibly due to the anti-inflammatory effects [117]. The NUTRAOLEUM study in Spain evaluated the pharmacokinetics following olive oils (OO) consumption with a high and triterpene content [118]. The enrichment of OO with the triterpenes (OA and MA) improved endothelial function, which is vital as endothelial dysfunction is considered an early sign of atherosclerosis [118]. These effects were mainly attributed to MA due to its high bioavailability compared to OA [118]. There is indeed a paucity of clinical trial studies evaluating MA and MA derivatives as antidiabetic compounds. 

### 3.4. Asiatic Acid (AA)

**Figure 6:**
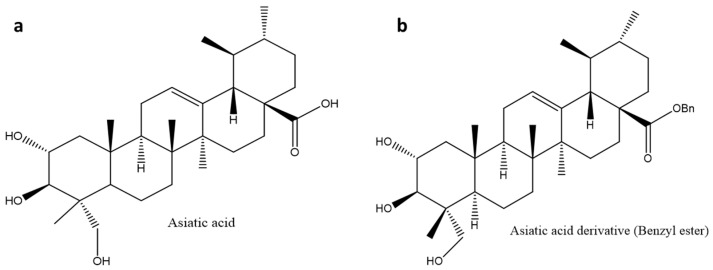
Chemical structure of (**a**) asiatic acid and (**b**) asatic acid derivative (benzyl ester). The asiatic acid derivative (**b**) was synthesized by incorporating a Benzyl ester at Carbon 28. This improved the anti-glycogen phosphorylase activity up to 4.5 times, thus enhancing antidiabetic properties.

Asiatic acid is a pentacyclic triterpenoid naturally found in *Centella asiatica* in two forms; free triterpene and asiaticoside (an active saponin). This compound has shown anti-diabetic, anti-alzheimer, anti-angiogenic, anti-inflammatory, anti-hepatitis C virus (HCV), antioxidant, anti-cancer, hepatoprotective, and neuroprotective activities [119,120,121]. Asiatic acid has been made commercially available under the trade name Madecassol® in Canada and some European Union countries due to its dermatological properties [122]. This triterpene (Figure 6a) has shown anti-cancer potential by inducing apoptosis and suppressing cancer cell proliferation and modulating apoptosis regulators such as glioblastoma multiforme (GBM), B-cell CLL/lymphoma 2 (BCL-2), and caspases [121]. The analgesic and anti-inflammatory effects of asiatic acid have been reported [123]. The overexpression of antioxidant enzymes such as superoxidase dismutase, catalase, glutathione peroxidase, and inducible nitric oxide synthase may be linked to its mechanism of action [121]. Another report suggested that asiatic acid’s anti-inflammatory activity might be associated with its inhibitory effects on cyclooxygenase-2 (COX-2), interleukin-6 (IL-6), interleukin-1 (IL-1), and tumor necrosis factor α (TNF-α) [124].

Asiatic acid has shown anti-diabetic and anti-obesity activities by enhancing insulin secretion and reducing the production of fatty acids in adipose tissue, respectively. The effect of asiatic acid on body weight is connected to its ability to inhibit the activity of pancreatic lipase and amylase, thereby driving weight loss [125]. Asiatic acid has been reported to exhibit anti-diabetic activities by inhibiting the production of free radicals linked to the development of diabetes embryopathy, nephropathy, and neuropathy [95,126,127]. Asiatic acid has been reported to lower blood glucose levels in STZ-induced diabetic rats. Furthermore, in STZ-induced diabetic rats, asiatic acid has been shown to considerably increase insulin levels, decrease lipid peroxidation, and improve the antioxidant system. This triterpene has been shown to improve glucose absorption in insulin-deficient STZ diabetic rats’ skeletal muscles [128]. A study reported better anti-carcinogenic and apoptotic activities of asiatic acid when loaded in solid lipid nanoparticles [129]. Asiatic acid’s cytotoxic efficacy against neoplasm P388D1 and melanoma Malme-3M cells has been investigated, resulting in better anti-cancer activity [130,131].

Although asiatic acid and its derivatives have shown a wide spectrum of biological actions, their medicinal effects remain limited due to poor bioavailability [121]. The authors of [132] investigated the bioavailability of asiatic acid. The study found that asiatic acid is rapidly metabolized by liver enzymes in rats, implying that it has a low oral bioavailability. Another study that evaluated the oral bioavailability of asiatic acid and asiaticoside in 12 healthy male and female adults, found that asiatic acid had a quicker maximal blood concentration. In contrast, asiaticoside had a greater sustained bioavailability. This further suggested that Madecassol®’s quick and long-lasting activity is due to the combined therapeutic effects of asiatic acid and asiaticoside [133]. To date, no clinical trials have been conducted on asiatic acid as an anti-diabetic agent. 

#### Glycogen Phosphorylase Inhibitor Derivatives

Many natural triterpenes and their derivatives have been known to exert their hypoglycaemic effects by inhibiting glycogen phosphorylase, α-amylase, and α-glucosidase. At least six possible regulatory binding sites have been identified in GP, and several structurally modified GP inhibitors have been identified and studied to improve the dissolution [18]. The study by [18], which investigated the inhibitory activity of asiatic acid (IC_50_: 17 µM) on GP, was followed by the synthesis and evaluation of 24 derivatives on GP. In some cases, the addition of a hydrophilic group at carbon-28 had no positive effect on the synthesized compounds, which explains the hydrophobic nature of most pentacyclic triterpenes. However, among the synthesized compounds, the asiatic acid benzyl ester (Figure 6b, Table 1) had the highest inhibitory activity (IC_50_: 3.8 µM) on GP when compared to other tested derivatives, and the lead compound with IC_50_ of 17 µM [95]. Furthermore, the structural activity relationship study of the derivatives revealed that asiatic acid with a 2α-OH function had greater activity against GP than the derivative with a 2β-OH function [134]. Although research on the structural modification of pentacyclic triterpenes to improve the solubility is interesting, the formulation of nanoemulsions to enhance the bioavailability and efficacy of these hydrophobic compounds needs to be considered.

### 3.5. Ursolic Acid (UA)

**Figure 7:**
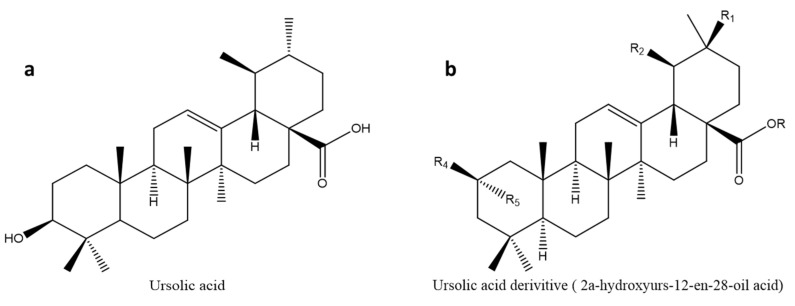
Chemical structure of (**a**) ursolic acid and (**b**) ursolic acid derivative (2α-hydroxyurs-12-en-28-oic acid). The ursolic acid derivative (**b**) exhibited a 17-fol inhibition of the glycogen phosphorylase. The antidiabetic effects are attributed to the migration of 3-OH to Carbon-2 rather than incorporating hydrophobic groups at Carbon-28.

Ursolic acid is a naturally occurring triterpenoid molecule found in foods, fruits, and plants. Rosemary, lavender, apple fruit peel, organum, thyme, berries, and flowers have all been found to contain ursolic acid. Ursolic acid has been shown to exhibit a variety of biological actions, and it is considered the most promising triterpene of the triterpenoid family. This compound (Figure 7a) has shown anti-cancer, anti-diabetic, hepatoprotective, anti-inflammatory, anti-obesity, cardioprotective, antioxidant, and anti-apoptotic properties and has been utilized as a component in health products and cosmetics because it is moderately non-toxic [85,135,136,137,138]. Ursolic acid has anti-cancer characteristics by modulating apoptotic signalling in cancer cells, suppressing carcinogenesis, and assisting in the clearance of damaged cells [139,140,141].

An ursolic acid derivative from Cynomorii Herba reduced the weight of high-fat diet-induced obese mice, possibly through the reduction of blood glucose levels [142]. Furthermore, ursolic acid and its supplements have shown anti-diabetic properties by inhibiting pancreatic α-amylase and uncoupling protein 3/AMPK-dependent pathways, resulting in reduced body weight and free fatty acid levels in high-fat obese rats [143,144,145]. Another research examined how ursolic acid affected some patients’ body weight and glucose tolerance, finding that it reduced body weight, body mass index, and insulin resistance [146]. This is relevant since increased body weight contributes to the development of DM. When ursolic acid was encapsulated in nanoemulsions, refs. [147,148] found that its anti-inflammatory, permeability, and anti-carcinogenic activities were enhanced. Despite the wide range of biological activities of ursolic acid, it is poorly soluble in water and has poor bioavailability [85].

#### 3.5.1. Glycogen Phosphorylase Inhibitor Derivatives

Since studies have shown that lowering hepatic glucose output can help control hyperglycemia, several GP inhibitors have been designed and evaluated for the treatment of type 2 diabetes [96]. The GP inhibitory activities of some synthetic compounds including 2α-hydroxyurs-12-en-28-oic acid (Figure 7b, Table 1) were evaluated. The 3-deoxy-2-keto derivative with IC_50_ of 24.2 µM showed less inhibitory activity on rabbit muscle GPa when compared to ursolic acid, which had IC_50_ of 15.3 µM [96]. Interestingly, in the same series of 35 synthesized 3-deoxypentacyclic compounds, 2α-isomer of 2-isoursolic acid (2α-hydroxyurs-12-en-28-oic acid) had the highest activity with IC_50_ of 1.2 µM when compared to other synthesized compounds and the lead compound. Structural–activity relationship study showed that displacement of the 3-OH group to carbon-2 of pentacyclic triterpenes may enhance GP inhibition as observed in 2-isoursolic acid (IC_50_: 5.5 µM) compared to ursolic acid with IC_50_ values of 15.3 µM. Furthermore, the addition of hydrophobic groups at carbon-2 and carbon-28 may not be effective for improving GP inhibition, making the nanoemulsions a suitable approach for enhancing the bioavailability of these compounds. 

#### 3.5.2. Clinical Studies on Ursolic Acid 

A randomized, double-blind, placebo-controlled clinical trial was performed on 24 patients between 30 and 60 to investigate the effects of UA (150 mg) on metabolic syndrome, insulin sensitivity and inflammation [146]. The inflammatory markers, interleukin-6 and C-reactive protein were analyzed using the ELISA technique [146]. The administration of UA resulted in the remission of metabolic syndrome by lowering body weight, waist circumference, fasting glucose, and insulin sensitivity [146]. However, study showed no effects of UA on inflammatory markers. On the contrary, a randomized clinical trial done on postmenopausal women (n = 61) indicated that supplementation of UA (450 mg/day) combined with physical exercise had no effects on metabolic syndrome parameters [149]. Although metabolic syndrome parameters were not affected, an 8-week treatment with UA improved systolic blood pressure, insulin resistance, and HOMA-IR [149]. From these clinical trials, it is evident that UA has moderate anti-diabetic properties in humans, posing translational discrepancies from animal studies. 

### 3.6. Corosolic Acid (CA)

**Figure 8:**
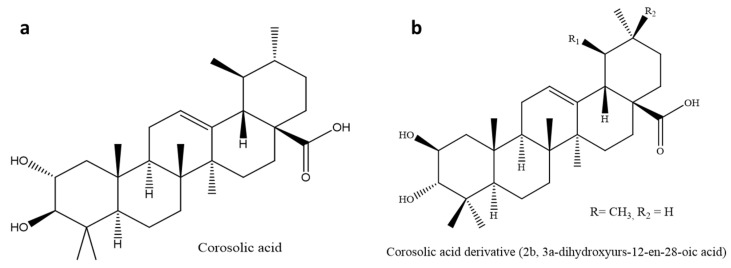
Chemical structure of (**a**) corosolic acid and (**b**) corosolic derivative 2β, 3α-Dihydroxyurs-12-en-28-oic acid. The corosolic acid derivative (**b**) was synthesized as a natural 2,3R-isomer of corosolic acid. This derivative improved the efficacy of the parent compound as glycogen phosphorylase inhibitor up to 18 times.

Corosolic acid, also known as 2α-hydroxyursolic acid, is a ursane-type triterpenoid found in *Lagerstroemia speciosa* L, *Eriobotrya japonica*, *Weigela subsessilis*, *Potentilla discolor* Bunge, *Orthosiphon stamineus*, and *Schisandra chinensis* [143,150,151]. This compound (Figure 8a) is known to regulate several biological processes in colorectal, cervical, and ovarian cancer through the activation of kinases and biological oxidative damage [151,152,153].

A decrease in fasting and postprandial blood glucose levels in human subjects has been reported to corroborate corosolic acid’s anti-diabetic activity [154,155]. Corosolic acid had the highest activity on α-glucosidase compared to oleanolic acid, arjunolic acid, asiatic acid, maslinic acid, and 23-hydroxyursolic acid [156]. Another anti-diabetic study evaluated two extracts of Lagerstroemia speciosa leaves containing 1% corosolic acid (Glucosol) in a type 2 diabetes mellitus clinical trial for two weeks. The results showed a 30% decrease in blood glucose levels when the soft gel was administered compared to 20% of the hard gelatin. This shows that the soft gel formulation had better bioavailability than the powdered formulation [157]. Furthermore, animal models evaluated the effects of corosolic acid on hypercholesterolemia and hepatic steatosis in type 2 diabetes. The results showed that corosolic acid had a 32% and 46% inhibitory effect on the mean blood and liver cholesterol levels, respectively [158].

#### 3.6.1. Glycogen Phosphorylase Inhibitor Derivatives

Since early studies have reported corosolic acid to be a natural inhibitor of GP, recent studies are increasingly focusing on identifying and developing structural GP inhibitors. A diastereoisomer of corosolic acid, 2β,3α-dihydroxyurs-12-en-28-oic acid (Figure 8b, Table 1), has been reported to have a higher GP inhibitory activity with IC_50_ of 1.1 µmol/L compared to corosolic acid (IC_50_: 20 µmol/L). Structural–activity relationship study suggested that the configuration of the 2,3-dihydroxy A-ring possibly improved the GP inhibitory activity favoring the 2β,3α-configuration than the 2α,3β-configuration [17,18]. Another study on the structural modification of corosolic acid, which majored on the carbon-24 A-ring, reported the significant inhibitory activities of three corosolic acid derivatives on rabbit muscle GPa with IC_50_ of 3.26, 5.1, and 7.31 µM, compared to the parent compound (IC_50_: 20 µM) [159]. 

#### 3.6.2. Clinical Studies on Corosolic Acid (CA)

Fukushima et al. conducted the first double-blinded study to investigate antidiabetic effects of corosolic (CA) in humans [97]. This study consisted of 31 participants divided into placebo and CA treated group (10 mg) for a 3-hour oral glucose tolerance test (OGTT). CA reduced blood glucose concentrations from 60 to 120 minutes and showed statistical significance at 90 minutes, thus corroborating the hypoglycemic effects seen in streptozotocin-induced diabetic rats at higher doses [97,160]. The moderate hypoglycemic impact of CA on human clinical trials indicate the need to consider derivatives such as 2β, 3α-Dihydroxyurs-12-en-28-oic acid) as a potential anti-diabetic derivative with improved efficacy. 

Table 1 indicates the structural activity relationship of the derivatives of some pentacyclic acids. In drug formulation, water solubility and bioavailability of bioactive compounds are crucial in enhancing drug efficacy. While most pentacyclic triterpenes are poorly soluble in water, their synthesized derivatives, by the addition of a hydrophobic side chain in most cases, have displayed high water-solubility and improved efficacy, as shown by the derivatives’ inhibitory effect on GP when compared to the parent pentacyclic triterpenes. 

## 4. Conclusions

Indeed, pentacyclic triterpenes have received much attention considering their wide range of therapeutic properties, particularly anti-diabetic. This review broadens our understanding of the work done so far to improve the efficacy of pentacyclic triterpenes in the control of blood glucose levels and related complications. The effort to synthesize pentacyclic triterpenes has mostly ended in in vitro studies where effective derivatives are not taken further into animal studies and then translated to human trials. There are two categories of derivatives presented in this review, those that are glycogen phosphorylase inhibitors and those that are not. Improving the efficacy of triterpenes as phosphorylase inhibitors requires the insertion of hydrophobic compounds, thus worsening solubility and efficacy. Bioavailability is particularly an essential issue in pentacyclic triterpene research, as the triterpenes act on the brush border and target the liver, kidney, and skeletal muscles. We, therefore, propose a solution to develop lipophilic nanoparticles for both oral and dermal delivery of the triterpenes to improve solubility, absorption, and efficacy. The derivatives that are not phosphorylase inhibitors have shown promising results. Bardoxolone methyl has been an extraordinary derivative with improved kidney ameliorative effects compared to the parent compound OA. While there are unexplainable findings, animal and cell culture work can substantiate mechanisms behind human clinical trials. To improve the translational research, it is recommended that researchers do a complete report on both positive and negative effects of the drugs underdevelopment from the invitro and animal testing stage. Toxicity studies should include the impact of the drugs on the QTc intervals and NRF-2 expression on rodents before human trials. Therefore, further studies should investigate the hindrance behind translating animal research to human clinical trials and how to improve the delivery of hydrophobic triterpenes and related derivatives to improve efficacy. 

## Figures and Tables

**Table 1:** Bioavailability and Efficacy of Some Pentacyclic Derivatives.

Derivatives	Structural Activity Modification	Activity	IC_50_ from Lead Compound to Derivative	Bioavailability	Reference
Oleanolic acid derivative (3β-{2-[4-(2-naphthalen-1-yl) acetoxymethyl-1H-1,2,3-triazol-1-yl] acetoxy} olean-12-en-28-oic acid	GP inhibitor	Reduces hepatic glucose synthesis	14 to 5.4 µM	N/A	[91]
Oleanolic acid-derivative (dipeptide diester prodrug)	N/A	Increased stability and permeability	N/A	enhanced	[92,93]
Maslinic acid-derivative ( 1,4-dibromo-butane at carbon 28)	GP inhibitor	Lowers blood glucose levels	28 to 7 µM	N/A	[94]
Asiatic acid-derivative (Benzyl ester)	GP inhibitor	Lowers blood glucose levels	17 to 3.8 µM	N/A	[18,95]
Ursolic acid-derivative (2α-hydroxyurs-12-en-28-oic acid)	GP inhibitor	Reduces hepatic glucose synthesis	15 to 1.2 µM	N/A	[96]
Corosolic acid-derivative (2β, 3α-Dihydroxyurs-12-en-28-oic acid)	GP inhibitor	Reduces hepatic glucose synthesis	20 to 1.1 µM	N/A	[17,18]
Corosolic acid-derivative (CO(CH_2_)_4_CH at carbon 2, H at carbon 3, H at carbon 28)	GP inhibitor	Reduces hepatic glucose synthesis	20 to 3.26 µM	N/A	[97]

GP: Glycogen phosphorylase; N/A: Not available.

## Data Availability

Not applicable.
